# Exosomes: A novel insight into traditional Chinese medicine

**DOI:** 10.3389/fphar.2022.844782

**Published:** 2022-08-29

**Authors:** Chao Mo, Jie Zhao, Jingyan Liang, Huiling Wang, Yu Chen, Guodong Huang

**Affiliations:** ^1^ Graduate School, Guangxi University of Chinese Medicine, Nanning, China; ^2^ Department of Nephrology, The First Affiliated Hospital of Guangxi University of Chinese Medicine, Nanning, China; ^3^ Graduate School, Hunan University of Chinese Medicine, Changsha, China; ^4^ Department of Nephrology, Guangxi International Zhuang Medicine Hospital Affiliated to Guangxi University of Chinese Medicine, Nanning, China

**Keywords:** traditional Chinese medicine, exosome, modulator, delivery carrier, biomarker

## Abstract

Exosomes are small extracellular vesicles and play an essential role in the mediation of intercellular communication both in health and disease. Traditional Chinese medicine (TCM) has historically been used to maintain human health and treat various diseases up till today. The interplay between exosomes and TCM has attracted researchers’ growing attention. By integrating the available evidence, TCM formulas and compounds isolated from TCM as exosome modulators have beneficial effects on multiple disorders, such as tumors, kidney diseases, and hepatic disease, which may associate with inhibiting cells proliferation, anti-inflammation, anti-oxidation, and attenuating fibrosis. Exosomes, a natural delivery system, are essential in delivering compounds isolated from TCM to target cells or tissues. Moreover, exosomes may be the potential biomarkers for TCM syndromes, providing strategies for TCM treatment. These findings may provide a novel insight into TCM from exosomes and serve as evidence for better understanding and development of TCM.

## Introduction

Intercellular communication is an essential hallmark of multicellular organisms to exchange information, which can be mediated via direct cell-to-cell contact or transfer of secreted molecules (Lee et al., 2012). Exosomes are small extracellular vesicles with a diameter of 30–150 nm involved in complex intercellular communication, which present in various biological fluids, such as blood, saliva, and urine (Andaloussi et al., 2013; Zabeo et al., 2017; Kalluri and LeBleu, 2020). Exosomes can be generated in the endolysosomal and multivesicular body compartments by most cells and secreted into the extracellular environment (Raposo and Stoorvogel, 2013). The process of exosome generation is associated with plasma membrane invagination, early-sorting and late-sorting endosome formation, multivesicular bodies construction, and outer membrane with the cellular plasma membrane fusion (Kalluri and LeBleu, 2020). Exosomes carry multiple intracellular signals, such as nucleic acids, proteins, lipids, and metabolites, for information and material exchange between cells, thus influencing their phenotype and cellular processes (Pegtel and Gould, 2019). Recipient cells uptake exosomes through cellular recognition, internalization, and cellular response (McKelvey et al., 2015; Mathieu et al., 2019).

Several studies demonstrated the pivotal role of exosomes in the diagnosis and treatment of multiple diseases, such as tumor diseases (Nam et al., 2020), metabolic disorders (Isaac et al., 2021), cardiovascular dysfunction (Barile et al., 2017), neurological injury (Zhang et al., 2019), and kidney disease (Jin et al., 2021). Notably, Exosomes, a bright star in drug delivery, have attracted researchers’ attention. Exosomes deliver nucleic acids, proteins, and small molecule drugs to target cells or tissues (Ferreira et al., 2022). Moreover, exosomes also enhance blood-brain barrier penetration (Liang L. et al., 2021). Collectively, exosomes hold significant promise with regard to biomarkers, therapies, and specific targeting.

Recently, exosome has become a hot spot in traditional Chinese medicine (TCM). The interplay between exosomes and TCM draws public attention increasingly. Growing evidence showed that TCM formulas, Chinese medicine monomers, or compounds isolated from TCM exhibit their effects on various diseases via modulating exosomes (Wei et al., 2016; Zheng et al., 2018; Liu et al., 2020; Wang X. J. et al., 2021). Moreover, exosomes may be the potential delivery carriers for compounds isolated from TCM (Dong M. et al., 2021a). TCM syndromes are closely associated with exosomes (He et al., 2021). These results demonstrate that exosomes may be a novel insight into TCM. The focus of this review is the interplay between exosomes and TCM.

## Search strategy

We systematically searched the literature in electronic databases, including PubMed, Embase, Cochrane Library, Web of Science, and Clinical Trials.gov, from inception to 30 November 2021. Medical subject heading terms and free words were as search terms, including “exosome”, “extracellular vesicles”, “medicine, Chinese traditional”, “traditional medicine Chinese”, “Chinese herbal medicine”, etc. We also manually screened the reference list of eligible studies in case of missing appropriate studies. The detailed search strategies are described in Supplementary File S1. Articles were limited to English publications. Studies related to exosomes and TCM *in vivo* and/or *in vitro* were included. Reviews and conference abstracts were excluded.

**FIGURE 1:**
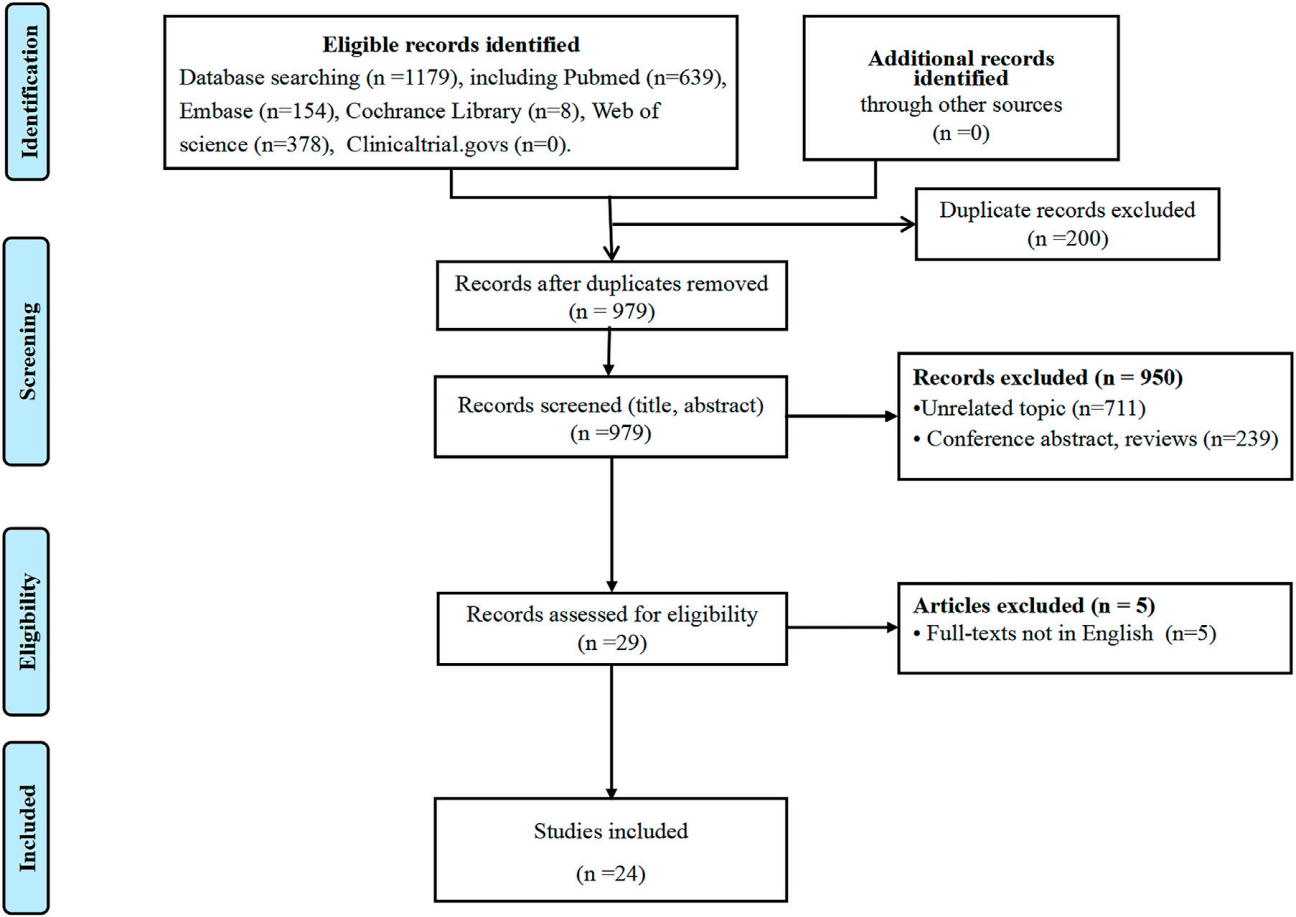
Flow diagram of the search for eligible studies.

A total of 1,179 citations from different databases were imported to the software Endnote X9. Literature was independently screened by two reviewers (CM and JZ) according to the inclusion and exclusion criteria. The full texts of eligible articles were further screened by the reviewers. Disagreements were resolved through discussion and consultation with a third investigator (GDH). Ultimately, 24 studies were included to further analysis, published from 2016 to 2021. The flow diagram is shown in Figure 1. Data, including the author’s name, year of publication, type of studies, characteristics of exosomes, effects, potential mechanisms, etc., were compiled in Tables 1–4.

**TABLE 1:** Exosomes modulators from TCM formulas on various diseases.

Study ID	Model	N	TCM interventions/dosage	Control interventions/dosage	Length of study	Characteristics of exosomes					Results	Effects/potential mechanisms
						Source	Isolation procedure	Morphology	Size distribution	Membrane surface markers		
Wang X. J, et al. (2021)	HUA patient	30	GGQLD: Puerariae Lobatae Radix (Gegen), Scutellariae Radix (Huangqin), Coptidis Rhizoma (Huanglian), and Glycyrrhizae Radix et Rhizoma Praeparata cum Melle (Gancao), in the ratio of 8:3:3:2. dosage: two doses daily	None	4 weeks	Urine	Manufacturer’s kit	Not report	Not report	CD63, TSG101	GGQLD decreased NLPR3 protein expression in urinary exosomes	GGQLD against HUA by inhibiting the mitochondrial apoptotic pathways through caspase-9, caspase-8, and Bcl-2/Bax/caspase-3, thus alleviating inflammation via inhibition of NLRP3/caspase-1/IL-1β/GSDMD.
Liu X. et al. (2020)	CKD rat	18	JPYSF: Astragali Radix (Huangqi), Atractylodis Macrocephalae Rhizoma (Baizhu), Dioscoreae Rhizoma (Shanyao), Cistanches Herba (Roucongrong), Amomi Fructus Rotundus (Baidoukou), Salviae Miltiorrhizae Radix et Rhizoma (Danshen), Rhei Radix et Rhizoma (Dahuang), and Glycyrrhizae Radix et Rhizoma Praeparata cum Melle (Gancao), in the ratio of 30:10:30:10:10:15:10:6. dosage: 10.89 g/kg/d	Equal volumes of distilled water	6 weeks	Serum	Manufacturer’s kit	Spherical structure	50–150 nm	TSG101, CD81, CD9	JPYSF upregulated the expression of exosomal miRNAs miR-192-5p	JPYSF plays a role in alleviating renal fibrosis in CKD rats, likely associated with regulating the exosomes expression
Zha et al. (2021)	EAE mouse	40	BSYSC: Rehmanniae Radix (Shengdihuang), Rehmanniae Radix Praeparata (Shudihuang), Polygoni Multiflori Radix (Heshouwu), Rhei Radix et Rhizoma (Dahuang), Leonuri Herba (Yimucao), Fritillariae Thunbergii Bulbus (Zhebeimu), Hirudo (Shuizhi), Scorpio (Quanxie), Gastrodiae Rhizoma (Tianma), and Forsythiae Fructus (Lianqiao), in the ratio of 10:10:10:2:10:6:3:2:3:6. dosage: 3.02 g/kg/once a day	Fingolimod dosage 0.3 mg/kg/once a day	40 days	Serum	ExoRNeasy Serum Mid Kit	Cup-shaped morphology	80–200 nm	CD63, HSP70, TSG101	BSYSC increased exosomal miR-124 expression and decreased exosomal miR-155 expression	BSYSC alleviates the inflammatory response and ameliorates neurological function in EAE mice by promoting microglia phenotypic transformation toward M2 polarization by regulating their target genes C/EBPα-PU.1 and SOCS1
Zhao et al. (2020)	EAE mouse	8	BSYSC: Rehmanniae Radix (Shengdihuang), Rehmanniae Radix Praeparata (Shudihuang), Polygoni Multiflori Radix (Heshouwu), Rhei Radix et Rhizoma (Dahuang), Leonuri Herba (Yimucao), Fritillariae Thunbergii Bulbus (Zhebeimu), Hirudo (Shuizhi), Scorpio (Quanxie), Gastrodiae Rhizoma (Tianma), and Forsythiae Fructus (Lianqiao), in the ratio of 10:10:10:2:10:6:3:2:3:6. dosage: 3.02 g/kg/once a day	Prednisone acetate dosage: 6 mg/kg/once a day	40 days	Serum	Qiagen Kit	Spherical morphology	30–100 nm	Alix, CD9	BSYSC upregulated the level of neuropilin-1, GTX, and miR-146, and downregulated the expression of miR-16, let-7, miR-15, miR-98, miR-486, and miR-182 in serum exosomes	BSYSC exerts neuroprotective effects on EAE mice by facilitating remyelination via regulating neuropilin-1and GTX proteins and miRs expression in serum exosomes
Li H, et al. (2020)	IgAN rat	48	ZWT: Aconiti Lateralis Radix Praeparata (Fuzi), Poria (Fuling), Atractylodis Macrocephalae Rhizoma (Baizhu), Paeoniae Radix Alba (Baishao), and Zingiberis Rhizoma Recens (Shengjiang), in the ratio of 3:3:2:3:3. dosage: low-dose ZWT group (8.4 g/kg), high-dose ZWT group (16.8 g/kg), ZWT-EXO group (7.5 mg/kg)	Prednisone dosage:2 mg/kg	16 weeks	Hk-2 cells	Manufacturer’s kit	Classical round-shaped vesicle	Average at109 nm	CD9, CD81, CD63	ZWT reinforced the secretion of exosomes in HK-2 cells and renal tissue, increased NLRP3, p-p65, caspase-1 and IL-1b proteins’ levels, and enhanced the colocalization of NLRP3 and ASC	ZWT has an anti-inflammation effect on IgAN rats, which may be associated with ZWT regulating exosome secretion to restrain NF-kB/NLRP3 signaling pathway
Chen et al. (2019)	CCLM mouse	15	DHZCP: Rehmanniae Radix (Shudi), Paeoniae Alba (Baishao), Glycyrrhizae (Gancao), Radix Et Rhizome (Shandougen), Radix Rhei Et Rhizome (Dahuang), Scutellariae Radix (Huangqin), Persicae Semen (Taoren), Amygdalus Communis Vas (xingren), Tabanus (Mengchong), Hirudo (Shuizhi), Holotrichiae Larva (Qicao),Toxicodendri Resina (Ganqi), Eupolyphaga Seu Steleophaga (Tubiechong), in the ratio of 20:8:6:5:4:4:4:4:4:4:2:2. dosage: 9.6 mg/10 g/d	Cisplatin dosage:0.02 mg/10 g/d	Not reported	Serum	Manufacturer’s kit	Not report	Not report	Flotillin-1, HSP70, Alix	DHZCP repressed the expression of CCL2 in serum exosomes	DHZCP inhibits colorectal cancer liver metastasis, likely associated with blocking CCL2/CCR2 activation in the liver and suppressing the CCL2-mediated M2-skewing paradigm
Ruan et al. (2018a)	C-MSC	24	SXJXP: tetramethylpyrazine (Chuanxiong) and borneol (Bingpian), the ratio was not reported	Tetramethylpyrazine and borneol	48 h	C-MSC	Ultracentrifugation + filtration	Grape-like cluster	Average at 100 nm	CD63, Tsg101	SXJXP promotes exosome secretion from cardiac mesenchymal stem cells and upregulated the protein and mRNA levels of Rab27b	SXJXP promotes exosome secretion from C-MSCs via regulating the GTPase-dependent pathway
Ruan et al. (2018b)	HL-1 cell	12	SXJXP-containing exosomes	Tetramethylpyrazine- exosomes and borneol- exosomes	24 h	C-MSC	Ultracentrifugation + filtration	Pellet	Average at 100 nm	CD63, CD81, Tsg101	SXJXP-Exo increased H3K27me3 and PCNA levels and suppressed UTX mRNA expression	SXJXP-Exo promotes cardiomyocyte proliferation, likely associated with epigenetic chromatin remodeling in recipient cardiomyocytes
Wu et al. (2017)	HG-treated GEC	24	Tongxinluo capsule: Panax ginseng (Renshen), leech (Shuizhi), scorpio (Quanxie), cicada slough (Chantui), woodlouse (Tubiechong), centipede (Wugong), radix paeoniae rubra (Chishao), borneol (Bingpian), Santalum Album L.(Tanxiang), Dalbergiae Odoriferae Lignum (Jiangxiang), Olibanun (Ruxiang), Ziziphi Spinosae Semen (Suanzaoren), the ratio was not reported	30 mmol/L glucose	Not reported	GECs	Ultracentrifugation	Spherical structure	30–100 nm	CD9, CD63	TXLC inhibits exosomes secretion from HG-treated GECs and downregulates exosomal TGF-ß1 mRNA levels	TXL ameliorates renal fibrosis by impeding exosomal TGF-β1 transfer from GECs to glomerular mesangial cells by regulating the TGF-β1/Smad3 pathway

BSYSC, bushen yisui capsule; CKD, chronic kidney disease; CCLM, colorectal cancer liver metastasis; C-MSC, cardiac mesenchymal stem cell; CCL2, CC chemokine ligand-2; DHZCP, dahuang zhechong pill; EAE, experimental autoimmune encephalomyelitis; GGQLD, gegen qinlian decoction; GECs, glomerular endothelial cells; GMCs, glomerular mesangial cells; HUA, hyperuricemia; HG, high glucose; HK-2, human renal tubular epithelial cells; IgAN, IgA nephropathy; JPYSF, jianpi yishen formula; NLRP3, NLR family pyrin domain containing 3; SXJXP, suxiao jiuxin pill; SOCS1, suppressor of cytokine signaling 1; TXLC, tongxinluo capsule; ZWT, Zhen wu-tang.

## Traditional chinese medicine

TCM, an essential part of the traditional Chinese culture, is one of the oldest healing systems with a long history of more than 2,000 years, gaining more and more attention due to its unique theoretical bases and wealth of experience. TCM is characterized by the concept of organic wholeness and treatment based on syndrome differentiation, which differs virtually from the bases of western medicine. The theories of TCM consist of different parts, including an integral whole, two opposing principles (Yin and Yang), three essential substances (Chi, blood, and essence), a four-seasons theory (spring, summer, autumn, and winter), five elements (wood, fire, earth, metal, and water). Humans are an integral whole based on the theories of TCM, including five internal organs (heart, liver, spleen, lungs, and kidneys), six hollow organs (gallbladder, stomach, large intestine, small intestine, bladder, and san jiao), and primary and collateral channels that links all of them together.

According to the TCM theories, the state of Yin and Yang keeps a dynamic balance in healthy persons. Disease syndromes will occur if the balanced state is disturbed by the cause of external evils, such as six evil (wind, cold, fire, dryness, dampness, summer heat) or endogenous factors, e.g., seven emotions stimulating (happiness, anger, worry, anxiety, sadness, fear, and terror) (Chan and Ng, 2020). Notably, four diagnostic methods (inspection, listening and smelling, inquiry, as well as pulse-taking and palpation) are indispensable steps in diagnosing TCM syndrome before taking therapeutic measures, namely “Pattern Differentiation and Treatment” (Tang et al., 2008). With respect to treatment, TCMs, including Chinese herbal medicine, natural products, acupuncture, moxibustion, acupoint application, or physical exercise, e.g., Tai Chi, Qigong, Baduanjin, are used to adjust yin-yang disharmony and boost the individual’s endogenous healing ability. Encouragingly, the effects of TCM on complicated miscellaneous diseases have been gradually revealed in recent years. For example, Tu’ Youyou won the 2015 Nobel Prize for discovering Artemisinin extracted from the Chinese herbal medicine *Artemisia annua L.* (Tu 2016)*.* Artemisinin is essential for antimalarial treatment and brings light to malaria patients (Lyu H. N. et al., 2021). Although arsenic trioxide has been considered a poison since ancient times, it has significant effects on acute promyelocytic leukemia (Wang et al., 2020). Currently, as one of the strategies for the COVID-19 remedy, TCM has made meaningful and lasting contributions to treating COVID-19 (Lyu M. et al., 2021). Compared to western medicine alone, the combination of TCM and western medicine significantly improved clinical symptoms and reduced clinical deterioration and mortality in COVID-19 patients, indicating that TCM plays an irreplaceable role in the battle against COVID-19 (Huang et al., 2021; Zhao Z. et al., 2021 **).**

## Novel insight into understanding the connotation of TCM theory based on exosomes

TCM has established an abundant theoretical system. However, many theories of TCM cannot be accepted and interpreted by modern medicine due to the difference in origin and development background. With modern science and technology development, researchers commit themselves to seek TCM theory’s connotation and material basis. Cui et al. considered that the material basis of TCM theory should be characterized by uniqueness, specificity, and verifiability, which are indispensable (Cui et al., 2009). Uniqueness refers to the substance derived from a unique secretory source; specificity means that the substance plays an essential role in the physiological and pathological process of the secretory source and targeted goal; and verifiability can reflect the biological functions of the substance on the secretory source and targeted goal (Cui et al., 2009). The characteristics of exosomes, such as secretory source cells, powerful targeting, biological function, and biocompatibility, are consistent with the requirements of the material basis of TCM theory, indicating that exosomes may be the optimist material basis for understanding the connotation of TCM theory. For example, the TCM theory “kidney governs the bones and engenders marrow” can be explained by exosomes. Li et al. found that exosome-derived from HK-2 cells targeted osteoblasts and promoted their proliferation (Li et al., 2017), which provided a reference for further research on other TCM theories. TCM theories provide a hypothesis to study the interplay between exosomes and TCM, and exosomes offer a novel insight into the connotation and scientificity of TCM theories.

## Exosomes modulators from TCM formulas

Since exosomes involve in the pathogenesis of various diseases (Aghabozorgi et al., 2019; Jia et al., 2021; Jing et al., 2021; Luo and Yi, 2021 **),** investigating potential pharmaceutical agents with exosome-modulating ability has become a new field of drug. TCM formulas attract growing concern ascribe to their potential therapeutic in modulating exosomes.

TCM formula refers to a group of two or more medicines appropriately combined according to syndrome differentiation and treatment principle, which has significant effects due to an enormously complex chemical cocktail (Bu et al., 2020). TCM formulas have been widely used in the treatment of multiple diseases. However, its pharmacological mechanism has not been fully illustrated. More and more researchers sought to explore the role and underlying mechanism of TCM formulas on various diseases from the perspective of exosomes. Exosomes modulators from TCM formulas on various diseases are discussed as follows **(** Table 1 **)**.

### Gegen Qinlian decoction

Gegen Qinlian decoction (GGQLD), a classic TCM formula documented in <Shang Han Lun>, has been widely used to treat intestinal and metabolic diseases for hundreds of years in China (Lu et al., 2021). Wang et al. initiated a clinical trial to explore the anti-inflammatory effects of GGQLD on ameliorating hyperuricemia (HUA) patients (Wang Y. et al., 2021). Compared with before treatment, GGQLD significantly lowered serum uricemia levels and promoted urine uric acid excretion. Besides, GGQLD downregulated the NLR family pyrin domain containing 3 (NLRP3) expression in urinary exosomes and peripheral blood mononuclear cells. Furthermore, the protection of GGQLD against HUA was verified in Oxonic acid-treated rat models and UA-induced proximal tubular epithelial cells (PTECs). As a result, GGQLD significantly alleviated the expression of NLRP3, IL-1β, caspase-1/3/8, Gasdermin D (GSDMD), and Bcl-2 *in vivo* and *in vitro*. The results indicated that GGQLD exhibits an anti-inflammatory effect on HUA by inhibiting the NLRP3/caspase-1/IL-1β/GSDMD pathways and the mitochondrial apoptotic pathways. However, the study has not reported whether GGQLD affects the expression of exosomes in serum or plasma in HUA. Further studies are warranted to explore the molecular mechanisms of GGQLD against HUA via exosomes.

### Jianpi Yishen formula

Jianpi Yishen formula (JPYSF), a TCM formula with notable renoprotective, antioxidative, anti-inflammation, and antiapoptosis effects, has been widely used to treat chronic kidney diseases (CKD) for decades (Liu et al., 2018; Zhou F. et al., 2021). Liu et al. conducted a study to investigate whether the underlying mechanisms of JPYSF on CKD are related to serum exosomal miRNAs (Liu et al., 2020). In the CKD rat model, the levels of exosomal miR-192-5p, miR-194-5p, miR-802-5p, and miR-143-3p were markedly downregulated, while JPYSF treatment significantly reversed these expressions. Further identified by real-time PCR showed that only miR-192-5p was significantly restored in the JPYSF treatment group, and its ROC curve for the diagnosis of CKD was 77.8% after JPYSF treatment, indicating that exosomal miR-192-5p was the most potential target for CKD for JPYSF treatment and CKD diagnosis. miR-192 is one of the most abundant miRNAs in the kidney and contributes to alleviating renal fibrosis by inhibiting TGF-β/Smad3 signaling (Chung et al., 2010). Furthermore, miR-192-5p suppresses the mRNA of the β1 subunit of the Na+/K + -ATPase, exerting effects on renal handling of fluid balance on a high-salt diet (Mladinov et al., 2013). Nevertheless, the role and mechanism of miR-192-5p in JPYSF-treated CKD have not been clarified. Whether JPYSF plays a role in alleviating renal fibrosis via targeting miR-192-5p to inhibit the activation of TGF-β/Smad3 signaling and JPYSF keeps renal handling of fluid balance by targeting miR-192-5p to modulate Na+/K + -ATPase through in CKD warrants further exploration.

### Bushen Yisui capsule

Bushen Yisui capsule (BSYSC), a Chinese compound medicine comprising ten herbs, has significant effects on anti-neuroinflammatory and neuroprotective in multiple sclerosis and its animal model of experimental autoimmune encephalomyelitis (EAE) (Fang and Zhang, 2017; Zha et al., 2021). Zhao et al. conducted an experimental study to explore the potential roles of BSYSC in promoting remyelination via regulating exosomes in EAE mice (Zhao et al., 2020). Compared with normal mice, EAE mice had a significant increase in neuropilin-1 and GTX expressions in serum exosomes, while BSYSC could reverse the phenomenon in EAE mice. Besides, the serum exosomal miR-146 level in EAE mice treated with BSYSC markedly upregulated, while the expression of miR-16, let-7, miR-15, miR-98, miR-486, and miR-182 decreased. The disequilibrium of Neuropilin-1 and GTX led to remyelination failure (Boyd et al., 2013), indicating that BSYSC facilitates remyelination via regulating neuropilin-1and GTX proteins and miRs expression in serum exosomes, thus exerting neuroprotective effects. However, the mechanism of BSYSC regulating the expression of neuropilin-1and GTX proteins and miRs in serum exosomes and whether BSYSC plays a direct or indirect role in the regulation of remyelination via exosomes has not been elucidated, warranting further exploration.

Another experimental study was conducted by Zha et al. to investigate the potential roles of BSYSC on microglial polarization in EAE mice. Compared with EAE mice, serum exosomal miR-155 expression was markedly increased, accompanied by downregulating exosomal miR-124 expression in EAE mice treated with BSYSC. Furthermore, the levels of C/EBPα and PU.1 were downregulated after BSYSC treatment, while the suppressor of cytokine signaling 1 (SOCS1) expression was upregulated. C/EBPα and PU.1 are downstream target genes of miR-124. It has been shown that miR-124 alleviates EAE by skewing microglial polarization from the M1 phenotype to the M2 phenotype via targeting C/EBPα-PU.1 (Ponomarev et al., 2011). SOCS1 is a target gene of miR-155 and plays a critical role in promoting differentiation from the M1 to M2 (Whyte et al., 2011; Zhang et al., 2020). The findings indicated that BSYSC alleviates the inflammatory response and ameliorates neurological function in EAE through promoting microglia phenotypic transformation toward M2 polarization to modulate the expression of pro/anti-inflammatory factors, which may be associated with regulating the expression of miR-124 and miR-155 (Zha et al., 2021). However, the role of exosomal miR-124 and miR-155 in BSYSC-mediated microglia M2 polarization in EAE remains unclear, needing further studies to illuminate. Moreover, whether the effects of BSYSC-mediated microglia M2 polarization *in vitro* also requires to be verified in cell experiments.

### Zhenwu tang

Zhenwu Tang (ZWT) is a well-known prescription from Treatise on Febrile Diseases written by Zhongjing Zhang. It has been widely used in Parkinson’s and various kidney diseases, such as IgA nephropathy (IgAN), membranous nephropathy, and diabetic nephropathy (Cai et al., 2010; Li et al., 2011; Liu B. et al., 2019; Li J. et al., 2020). Li et al. implemented a study to investigate the potential mechanisms of ZWT on IgAN from the perspective of exosomes (Li J. et al., 2020). The rats were injected with exosomes isolated from HK-2 cells treated with 10% ZWT by tail vein as the ZWT-EXO group. Compared with the model group, CD63 fluorescence was notably intensified in the ZWT group, and the levels of CD63, CD81, and CD9 in the renal tissues were also dramatically upregulated in the ZWT group, indicating that ZWT reinforces the secretion of exosomes in IgAN rats. In addition, the protein levels of NLRP3, p-p65, caspase-1, and IL-1b were increased, and the colocalization of NLRP3 and ASC was highly expressed in the kidney tissues of IgAN rats, while these expressions were markedly reversed after following ZWT-EXO and ZWT treatment. Nuclear factor-kappa B (NF-κB) is an early responding regulator related to the expression of numerous proteins involved in inflammation (Wang et al., 2018). The transcription NF-kB promotes NLRP3 activation, which recruits ASC and caspase-1 proteins and leads to the release of IL-1b and IL-18, thus inciting phlogistic response (Ma et al., 2019). ZWT has an anti-inflammation effect on IgAN, which may be associated with ZWT regulating exosome secretion to restrain NF-kB/NLRP3 signaling pathway, thereby attenuating renal dysfunction. The cargoes in exosomes include proteins, miRNAs, long noncoding RNAs, mRNAs, and lipids; however, this study did not identify which one was regulated by ZWT. And whether ZWT inhibits the NF-kB/NLRP3 signaling pathway via regulating its downstream or upstream target genes in IgAN also needs further exploration.

### Dahuang zhechong pill

Dahuang Zhechong Pill (DHZCP) is a famous and classical formula from “Synopsis of Prescriptions of the Golden Chamber.” DHZCP has been widely used in the treatment of atherosclerosis, and gynecological disease, especially in hepatic diseases in China (Ji et al., 2007; Yuan 2009; Gong et al., 2020). To investigate whether DHZCP suppresses the metastasis of colorectal cancer, Chen et al. established a colorectal cancer liver metastasis model (Chen et al., 2019). Compared with the model group, DHZCP dramatically decreased serum exosomal CC chemokine ligand-2 (CCL2) level and its receptor CCR2. Exosomal CCL2 triggered macrophage recruitment and transformed the M1/M2 paradigm to a M2 phenotype in the liver; luckily, DHZCP treatment curbed the condition. CCL2/CCR2 is a prime factor in triggering fibrosis and promoting tumor metastasis by inducing macrophage accumulation (Fei et al., 2021; Klueh et al., 2016). M2 macrophages play critical roles in fibrosis, collagen synthesis, tissue remodeling, and pro-tumor processes (Mantovani et al., 2013; Braga et al., 2015; Shapouri-Moghaddam et al., 2018). The macrophage polarization states accelerate tumor growth, especially by skewing macrophages toward the M2 phenotype (Ricketts et al., 2021). The results indicated that DHZCP inhibits colorectal cancer liver metastasis, likely associated with blocking CCL2/CCR2 activation in the liver and suppressing the CCL2-mediated M2-skewing paradigm. However, further studies should focus on the mechanisms of DHZCP regulating CCL2/CCR2.

### Suxiao Jiuxin Pill

Suxiao Jiuxin pill (SXJXP), one of the Chinese patent medicines included in the Chinese Pharmacopeia, is well-known for notable cardioprotective effects (Ren et al., 2018). Ruan et al. studied the impact of SJP on exosome secretion in cardiac mesenchymal stem cells (C-MSCs) treated with SXJXP (Ruan et al., 2018a; Ruan et al., 2018b). SXJXP increased exosomes secretion from C-MSCs and upregulated the protein and mRNA levels of Rab27b; however, Rab27b knockdown inhibited exosome secretion. Rab27b is a small GTPase in the Rab family and controls exosome secretion (Ostrowski et al., 2010), indicating that SXJXP promotes exosome secretion from C-MSCs via regulating the GTPase-dependent pathway. Results of another study showed that SXJXP-Exo increased histone-3-lysine 27 trimethylation (H3K27me3) and proliferating cell nuclear antigen (PCNA) levels, accompanied by suppressing UTX mRNA expression in the HL-1 cells, a mouse cardiomyocyte line. H3K27me3, a critical epigenetic chromatin marker, promotes cell proliferation (Hansen et al., 2008). UTX is a demethylase of H3K27me3. The H3K27me3 demethylation is essential for the induction of direct cardiac reprogramming (Dal-Pra et al., 2017). Therefore, SXJXP-Exo promotes cardiomyocyte proliferation, likely associated with epigenetic chromatin remodeling in recipient cardiomyocytes.

### TongXinLuo capsule

Tongxinluo Capsule (TXLC) possesses a variety of pharmacological effects, including antihypertensive, improving ventricular remodeling, and antioxidant, protecting podocytes, which has beneficial effects on angina pectoris and CKD (Li et al., 2021; Mao et al., 2015). Wu et al. conducted an experimental study to investigate the role of TXLC on exosomes secretion from high glucose (HG)-treated glomerular endothelial cells (GECs) (Wu et al., 2017). Wu found that TXLC repressed the expression of TGF-β1-containing exosomes secretion from HG-Treated GECs. TGF-β1/Smad3 signaling has been considered a significant pathway in glomerular mesangial cells (GMCs) activation and renal fibrosis (Yu et al., 2022). Exosomes secretion from HG-Treated GECs dramatically increased the deposition and secretion of Col-IV and FN and upregulated TGF-β1 and phospho-Smad3 in GMCs. Luckily, TXLC curbed these phenomena. The findings suggested that TXL ameliorates renal fibrosis by impeding exosomal TGF-β1 transfer from GECs to GMCs by regulating the TGF-β1/Smad3 pathway. However, whether TXLC has beneficial effects on patients with diabetic kidney disease via the regulation of exosomes remains elusive.

## Exosomes modulators from compounds isolated from TCM

Natural products are compounds derived from various natural sources, such as plants, animals, and micro-organisms, which play a vital role in human health (Bernardini et al., 2018). It has been reported that natural products derived from TCM, such as Artemisinin, have significant effects on various diseases by modulating exosomes (Bai et al., 2019; Qiu et al., 2022). Compounds isolated from TCM are typically categorized as alkaloids, flavonoids, saccharides, saponins, terpenoids, and polyphenols based on their chemical property. Researchers have discovered the modulators of exosomes from compounds isolated from TCM and sought to clarify their mechanisms. The information on exosome modulators from compounds isolated from TCM is compiled in Table 2.

**TABLE 2:** Exosome modulators from compounds isolated from traditional Chinese medicine.

Study ID	Compounds	Herbs	Model	Characteristics of exosomes					Results	Effects/potential mechanisms
				Source	Isolation procedure	Morphology	Size	Membrane surface markers		
Ku et al. (2021)	Kaempferitrin	Cinnamomum osmophloeum (RouGui)	Liver cancer cell line HepG2 cells	HepG2 cells	Filtration and centrifugation	Not reported	Average at 64.72 nm	HSP90a, UBA1, HSP70	Kaempferitrin downregulated HSP90a proteinand and upregulated UBA1 protein in exosomes	HSP90AA1, HSP90AB1, and UBA1 may be exosomal markers of Kaempferitrin-treated HepG2
Bai et al. (2019)	Artemisinin	Artemisia annua L (QingHao)	Human mesangial cells	HK-2 cells	Manufacturer’s kit	Spherical morphology	20–175 nm	CD63, CD81	Artemisinin increased exosomes release and enhanced exosomes shuttling from HK-2 cells to human mesangial cells	Artemisinin has anti-inflammation and cells proliferation inhibition effects in human mesangial cells by reduced the inhibiting NF-κB/NLRP3 pathway via regulating exosomes secretion
Xia et al. (2019)	Halofuginone	Dichroa febrifuge (ChangShan)	Human breast cancer MCF-7 cells	MCF-7 cells	Manufacturer’s kit	Round or oval membranous vesicles	11–100 nm	Tsg101, CD63, CD81,CD9	Halofuginone repressed exosomal miR-31 secretion	Halofuginone inhibites cells proliferation by repressing exosomal miR-31 secretion and delivery by modulating the HDAC2/cell cycle signaling axis
Wei et al. (2016)	Shikonin	Lithospermum erythrorhizon (ZiCao)	Human breast cancer MCF-7 cells	MCF-7 cells	Manufacturer’s kit	Double membrane vesicles	50–100 nm	CD63, Tsg101, CD9, GAPDH	Shikonin inhibited exosomal miR-128 secretion	Shikonin inhibites cells proliferation, likely associated with reducing tumor-derived exosomal miR-128 by targeting the Bax gene

HSP90AA1, Heat shock protein HSP 90-α; HSP90AB1, Heat shock protein HSP 90-β; HDAC2, histone deacetylase 2; NLRP3, NLR family pyrin domain containing 3; UBA1, Ubiquitin-like modifier-activating enzyme 1.

### Kaempferitrin

Kaempferitrin, a potent flavonoid compound derived from the leaves of *Cinnamomum osmophloeum*, is well-known for its anti-diabetic, antioxidant, and anti-inflammatory properties (Li et al., 2013; Jiang et al., 2018; Wang et al., 2019). Ku et al. explored the effects of Kaempferitrin on HepG2 differentially expressed exosomes and extracellular vesicle sizes (Ku et al., 2021). The proteins expressions of Heat shock protein HSP 90-α (HSP90AA1), Heat shock protein HSP 90-β (HSP90AB1), and Ubiquitin-like modifier-activating enzyme 1(UBA1) were differentially expressed in exosomes from Kaempferitrin-treated HepG2 group and the control group, of which HSP90a was downregulated and UBA1 was upregulated. The extracellular vesicle size was more significant in HepG2 cells treated with Kaempferitrin than in the control group. The results suggested that HSP90AA1, HSP90AB1, and UBA1 may be exosomal markers of Kaempferitrin-treated HepG2. And the extracellular vesicle sizes may be regulated by kaempferitrin. Kaempferol, a molecule with a structure similar to kaempferitrin, plays a crucial role in activating the membrane-bound ATPase, which provides energy during membrane budding from the cytoplasm (Al-Numair et al., 2015). Whether Kaempferitrin regulates the exosomal size and membrane assembly by modulating ATPase activities requires further exploration. Besides, the role and potential mechanism of exosome secretion and size regulated by Kaempferitrin on disease pathogenesis remain unclear, which will be an interesting direction to research.

### Artemisinin

Artemisinin, a sesquiterpene lactone isolated from *Artemisia annua L.* with outstanding antimalarial effect, has been regarded as the most effective and impactful antimalarial drug (Paddon et al., 2013). Except for its antimalarial effect, ART has exhibited effects on antioxidant, anti-inflammation, anti-fibrotic, and anti-tumor (Dolivo et al., 2021; Meng et al., 2021). Bai et al. conducted a study investigating whether Artemisinin could regulate inflammation factors by mediating exosome release *in vivo* (Bai et al., 2019). Artemisinin increased exosomes release in human renal tubular epithelial cells (HK-2) and enhanced exosomes shutting from HK-2 cells to human mesangial cells (HMCs). Mesangial cells are essential to maintain the normal function of the glomerulus, which play important roles in structural support and injury repair (Nowling 2022). The inflammatory states accelerate mesangial cells proliferation, a major contributor to glomerulosclerosis (Donegan et al., 2016). NF-κB, an upstream activator of the NLRP3 inflammasome, is known to regulate inflammatory processes in many diseases, including chronic kidney diseases (Lei et al., 2020). The protein expressions of IκB-α, p-p65, NLRP3, ASC, IL-1β, and caspase-1 were upregulated in HMCs, while these increased expressions were markedly curbed by Artemisinin. These findings suggested that ART has a positive anti-inflammation in HMCs, which may be associated with inhibiting NF-κB/NLRP3 pathway via regulating exosomes secretion, thereby ameliorating renal damage. However, the mechanisms of exosomes how to regulate the NF-κB/NLRP3 pathway have not been elucidated.

### Halofuginone

Halofuginone, a minor alkaloid extracted from the Chinese traditional herb *Dichroa febrifuge*, has significant anti-tumor, anti-hypertrophic, and anti-fibrotic properties (Pines and Spector, 2015; Jain et al., 2021). Xia et al. investigated the mechanism of Halofuginone inhibiting MCF-7 cell growth *in vitro* from the perspective of exosomes (Xia et al., 2019). As shown in the results, Halofuginone inhibited MCF-7 cell growth via repressing exosome secretion. Further miRNA profiles analysis showed that miR-100, miR-222, miR-31, miR-200, miR-223, and miR-21 were more abundant in exosomes secretion from MCF-7 cells treated with Halofuginone than in MCF-7 cells. And the inhibition of MCF-7 cell proliferation was strengthened only in the miR-31 knockdown exosomes, whereas miR-31 overexpression could attenuate Halofuginone-inhibited MCF-7 cell proliferation. Importantly, the level of HDAC2 was reduced by pre-miRNA of MCF-7-derived exosomal miR-31. HDAC2 siRNA repressed the level of cell cycle components in the MCF-7 cell’s G1/S transition, thereby inhibiting the MCF-7 cell’s growth. These findings suggest that Halofuginone inhibits MCF-7 cell proliferation by repressing exosomal miR-31 secretion and delivery by modulating the HDAC2/cell cycle signaling axis, thus exerting an anti-tumor effect. However, it remains to investigate whether Halofuginone inhibits tumor progress by modulating exosomal miR-31 *in vivo*.

### Shikonin

Shikonin, a naphthoquinone isolated from *Lithospermum erythrorhizon*, is well-known for its strong anti-tumor effects (Sun et al., 2022). It has been used to treat various cancers, such as colon, renal, and lung cancer (Pan et al., 2021; Shi G. et al., 2021; Tsai et al., 2021). To elucidate the roles and mechanisms of Shikonin on breast cancer, Wei et al. performed a study on MCF-7 cells with Shikonin treatment (Wei et al., 2016). Shikonin inhibited MCF-7 cell proliferation and exosome secretion from MCF-7 cells with a positive relationship. According to the results from analyzing miRNA profiles and qRT-PCR, exosomal miR-128 positively inhibited MCF-7 cell proliferation. Bax, a key regulator of the intrinsic or mitochondrial apoptosis pathway, has been reported as a target of miR-128 (Ji et al., 2013; Cosentino et al., 2022). Exosomal miR-128 significantly suppressed Bax expression in recipient MCF-7 cells. Therefore, Shikonin inhibits MCF-7 cell proliferation, which is likely associated with reducing tumor-derived exosomal miR-128 by targeting the Bax gene. However, the regulatory effects of Bax on the cell cycle modulation have not been clarified. Whether the mechanisms of Bax in MCF-7 cells proliferation is related to mitochondrial morphological changes or mitochondrial apoptosis pathway is also not well understood.

## Exosomes as delivery vehicles for compounds isolated from TCM

Chinese herbal medicine is composed of various compounds with multiple pharmacological effects. However, compounds isolated from TCM are confronted with obstacles in the application due to their poor water solubility, poor intestinal absorption, and low bioavailability, resulting in decreased therapeutic efficacy (Puglia et al., 2017). Thus, there is an urgent need for TCM compound-based drug delivery systems to overcome these constraints to maximize the performance of TCM in therapy. Exosomes are regarded as a natural delivery system due to their excellent biodistribution, biocompatibility, and low immunogenicity (Liao et al., 2019; Gutierrez-Millan et al., 2021). Recently, many studies have confirmed the feasibility of using exosomes for drug delivery in various diseases, exhibiting enhanced curative effects. For example, exosomes derived from dendritic cells delivered siRNA across the blood-brain barrier in wild-type mice, demonstrating the therapeutic potential of exosome-mediated siRNA delivery (Alvarez-Erviti et al., 2011). Doxorubicin-loaded exosomes showed great efficiency in breast cancer cells (Xie et al., 2021). Thus, exosomes as delivery vehicles for compounds isolated from TCM have become a hotspot in pharmaceutical research and therapeutics (Table 3).

**TABLE 3:** Exosome as delivery vehicles for compounds isolated from traditional Chinese medicine.

Study ID	Cargos	Exosome source	Loading strategies	Characteristics of cargo-containing exosomes					Target to	Targeting strategy	Main achievements
				Isolation procedure	Morphology	Size	Membrane surface markers	Others			
Han et al. (2019)	Icariin	Fetal bovine serum	Co-incubation	Ultracentrifugation	Typical lipid bilayer membrane structure	Average size at 122 nm	CD63, CD81, CD40	The efficiency of incorporation of Icariin in FBS EXO was about 13%	MC3T3-E1 cells	Co-incubation	Promoted the proliferation of osteoblasts and bone regeneration by increasing the expressions of osteogenic markers BMP-2, RUNX2, and OPN.
Liu H. et al. (2019)	Triptolide	Human ovarian cancer SKOV3 cells	Sonication	Ultracentrifugation and ultrafltration centrifugation	Ellipse shape	Average at 159.9 ± 2.7 nm	CD9, CD81	The encapsulation efciency of Triptolide in TP-Exos was 76.5 ± 1.8%	Human ovarian cancer cell lines SKOV3	Co-incubation	Attenuated the cytotoxic and apoptosis-inducing capability but enhanced the inhibition of cells proliferation of triptolide
									Mouse was subcutaneously injected with SKOV3 cells	Peritoneal injection	Enhanced anti-tumor effect of triptolide on ovarian cancer but had toxicity in liver and spleen
Wang and Zhao, (2019)	Curcumin	Murine macrophage RAW264.7 cells	Co-incubation	Ultracentrifugation	Round in shape	Average at 117.4 ± 10.5 m	Alix, CD63	The average encapsulation efficiency and loading capacity of Curcumin were 84.8% and 15.1%	SD rats	Intravenous injection	Increased the solubility, stability, and bioavailability of curcumin and facilitates higher accumulation of curcumin in the brain, with no significant toxicity
									Brain microvascular endothelial cell line (hCMEC/D3 cells)	Co-incubation	Enhanced cellular uptake and blood–brain barrier-crossing of curcumin via interacting between its inherited LFA-1 and endothelial ICAM-1
									Alzheimer’s disease mouse established by injecting okadaic acid	Intraperitoneal injection	Enhanced neuroprotection effect and attenuated cognitive decline by inhibiting phosphorylation of the Tau protein through activating the AKT/GSK-3β pathway
Abbasifarid et al. (2021)		HEK-293T cells	Sonication and freeze-thaw cycles	Centrifugation	Spherical structures	150–250 nm	CD63, CD9	The bsorption range from 1.78-1.8 (OD 410-430 nm)	C57BL/6 mouse that was subcutaneously injected with 0.1 × 106 TC-1 tumor cells	Intraperitoneal injection	Exerted anti-tumors effect by inducing T-cell immune responses and eradicating tumor cells
Fang and Liang, (2021)	Quercetin	Adipose mesenchymal stem cells	Co-incubation	ExoEasy Maxi Kit	Spherical structures	Not reported	Not reported	The binding capacity of Quercetin was 19.6%	Acute liver injury mouse established by injecting carbon tetrafluoride	Intravenous injection	Reduced acute liver injury by inhibiting rapid senescence-like response
Qi et al. (2020)		Plasma of SD rats	Ultrasonification	Not reported	Typical homogeneous and spherical vesicles	125–150 nm	Alix, CD63	The encapsulation efficiency of Quercetin at 30 ± 8.3% and drug loading ratio at 17.3 ± 6.3%	Alzheimer’s disease mouse established by injecting okadaic acid	Intraperitoneal injection	Enhanced bioavailability, improved brain targeting, and improved cognitive function by inhibiting phosphorylated tau-mediated neurofibrillary tangles
Fan et al. (2020)	Resveratrol	Primary microglia from the spinal cords of fetal rats	Co-incubation	Centrifugation	Normal morphological	Average at 113.4 ± 12.1 m	CD63, CD81, TSG101, GRP94	Not reported	Spinal cord injury rats	Not reported	Enhanced the solubility and stability of resveratrol and promoted motor functional recovery in spinal cord injury by activating autophagy and inhibiting apoptosis via inhibiting the PI3K signaling pathway
Liang Y. et al. (2021)	Norcantharidin	Bone mesenchymal stem cell	Electroporation	Ultrafiltration centrifugation	Saucer-like spherical structures	Average at 127 nm	Not reported	Not reported	Hepatocellular carcinoma cell lines hepG2 cells	Co-incubation	Promoted cellular uptake, induced cell cycle arrest, reduced tumor cell proliferation, increased apoptosis
									Hepatocellular carcinoma mice	Tail vein injection	No body toxicity; repaired damaged liver via increasing cellular proliferation and inhibiting oxidation of liver cell

### Icariin

Icariin, a prenylated flavonoid, is one of the main components of *Herba Epimedii* (Li et al., 2015). It has been reported that Icariin possesses various biological effects, including osteoporosis prevention, ameliorating sexual dysfunction, modulation of the immune system, improvement of cardiovascular function, anti-inflammatory, antioxidant, and anti-depressant (Fang et al., 2017; Angeloni et al., 2019). Although icariin has significant efficacy on diverse diseases, such as osteoporosis, the main challenge remains its very low solubility, permeability, and poor bioavailability (Han et al., 2019).

Fetal bovine serum (FBS)-derived exosomes as natural nanoscale carriers have been studied to deliver Icariin (Exo- Ica). The results showed that Icariin-containing exosomes significantly increased the proliferation of MC3T3-E1 cells and the protein levels of osteogenic markers Bone morphogenetic protein-2 (BMP-2), Runt-related transcription factor 2 (RUNX2), and Osteopontin (OPN) compared to those treated with Icariin and FBS exosomes, indicating that Exo-Ica effectively promotes the proliferation of osteoblasts and bone regeneration (Dong M. et al., 2021a). However, the mechanisms of cellular uptake of Exo-Ica and Exo-Ica on promoting osteoblast proliferation remain unknown. Runx2 is an important transcriptional factor involved in osteogenic differentiation, and its deletion leads to a disorder of bone formation (Takahata et al., 2021). AMP-activated protein kinase (AMPK) regulates the differentiation of osteoblasts and bone formation (Li et al., 2018). It has been demonstrated that the activation of the AMPK/Runx2 pathway is an important mechanism underlying the facilitating effects of agents against osteogenic differentiation in MC3T3-E1 cells (Wang Q. et al., 2022). Thus, whether Exo-Ica enhances osteogenic differentiation in MC3T3-E1 cells by regulating the AMPK-α/Runx2 pathway may be an interesting direction in the next studies.

### Triptolide

Triptolide, a natural diterpene triepoxide compound isolated from *Tripterygium wilfordii Hook F*, has promising multiple pharmacological activities, particularly anti-inflammatory, immunosuppressive and anti-tumor activities (Yuan et al., 2019; Gao et al., 2021). However, the application of TPL in the clinic is restricted due to its multiorgan toxicity and poor solubility (Hou et al., 2019).

Triptolide was loaded into exosomes derived from human ovarian cancer SKOV3 cells, and the effects of the triptolide-loaded exosomes (Exos-TP) delivery system on ovarian tumors was observed both *in vitro and in vivo* (Liu H. et al., 2019). The cytotoxicity and apoptosis-inducing capability of Exos-TP were weaker than that of free triptolide in SKOV3 cells, but the inhibitory effect of Exos-TP on cell proliferation was superior to free triptolide. Interestingly, Exos-TP significantly enhanced the inhibition of SKOV3 cells proliferation at 24 h but not at 48 h, likely associated with TP-Exo blocked cells in the S phase at 24 h and G0/G1 phase at 48 h. The effects of Exos-TP on proliferation and apoptosis of cells were confirmed *in vivo*, which was consistent with that *in vitro*. Besides, the tumor suppression of TP-Exo was significantly better than that of free triptolide *in vivo*. Although the pathological damage in the kidney, heart, lung, and ovary was not observed in all groups, the pathological damage in the liver was obvious in SKOV3 cells-derived exosomes group and TP-Exo group, and the spleen damage was in the free triptolide group and TP-Exo group, indicating that SKOV3 cells-derived exosomes in TP-Exo and triptolide in TP-Exo may be the cause of liver injury and spleen injury, respectively. Together, these findings suggested that TP-Exo can enhance the inhibitory effect of triptolide on tumor cell proliferation (Liu B. et al., 2019). However, the cytotoxicity of TP-Exo on liver and spleen injury needs to be further optimized to solve, and the underlying mechanisms of TP-Exo anti-tumor effects also require further study.

### Curcumin

Curcumin, a polyphenol compound extracted from the rhizomes of *Curcuma longa L.* (Zingiberaceae) (Kharat et al., 2017), has diverse pharmacological effects, including anti-tumor, anti-inflammatory, and antioxidative activities (Memarzia et al., 2021). However, its clinical application has been limited due to its poor water solubility and stability, bioavailability, and poor brain targeting.

Compared with free curcumin, exosomes derived from murine macrophage RAW264.7 cells with encapsulated curcumin (Exos-Cur) significantly improved the solubility, stability, and bioavailability *in vivo*. Besides, Exos-Cur enhanced cellular uptake and blood-brain barrier penetration via interacting with the inherited LFA-1 and endothelial ICAM-1 *in vitro* (Wang et al., 2019). Exos-Cur have great potential in enhancing neuroprotection and attenuating cognitive decline effects in Alzheimer’s disease therapy, as a study on Alzheimer’s disease mice showed that Exos-Cur markedly relieved the symptoms of Alzheimer’s disease by inhibiting phosphorylation of the tau protein via activating the AKT/GSK-3β pathway (Wang et al., 2019). A recent study supported that HEK-293T cells-exosomes loaded with curcumin significantly increased the levels of total IgG, IgG2a, IgG2b, IFNγ, Granzyme B, and lymphocyte proliferation in the C57BL/6 mice model injected with TC-1 tumor cells (Abbasifarid et al., 2021). The results indicate that the induction of T-cell immune responses eradicates tumor cells, thereby exerting anti-tumor effects. However, the cellular uptake mechanism of the exosomal curcumin has not been illustrated.

### Quercetin

Quercetin, a phenolic flavonol compound, can be extracted from multiple Chinese medicine herbs, including *Mulberry leaves, Radix Bupleuri*, *licorice, Astragali Radix, and Panax notoginseng* (Shi W. et al., 2021; Zhao J. et al., 2021; Chen et al., 2022; Tu et al., 2022). Accumulating studies have shown that quercetin has diverse pharmacological effects, including antioxidant, anti-aging, anti-fungal, anti-tumor, anti-inflammatory, anti-depressant, and hepatoprotective activities (Arabpour et al., 2021; Zou et al., 2021; Chen et al., 2022). However, it has not been fully harnessed in the clinic due to its low bioavailability.

A previous study has reported that exosomes derived from adipose mesenchymal stem cells (ASCs) show beneficial effects on liver diseases (Qu et al., 2017). Quercetin was encapsulated into ASCs-derived exosomes, and the effect of quercetin-loading exosomes on acute liver injury was studied (Fang and Liang, 2021). It was found that exosomal quercetin is more stable than free quercetin. Furthermore, the liver index, aging-related genes P16 and P21 levels, senescence-associated secretory phenotype markers IL-6, Ccl2, and Cxcl2 expressions were significantly reduced in the quercetin-laden exosomes group compared to the model group and the exosomes group, suggesting that quercetin-laden exosomes exhibit hepatoprotective effects on acute liver injury by inhibiting rapid senescence-like response and quercetin may enhance the therapeutic efficacy of ASCs-derived exosomes in liver disease (Fang and Liang, 2021). Nevertheless, whether the roles of quercetin in acute liver injury are enhanced by exosomes requires further research. Another study supported that exosomes loaded with quercetin not only significantly enhanced the bioavailability of quercetin and its accumulation in the brain region in SD rats but also improved cognitive function in Alzheimer’s disease mice by inhibiting the formation of insoluble neurofibrillary tangles by reducing cyclin-dependent kinase five mediated phosphorylation of tau protein (Qi et al., 2020), indicating exosomal quercetin as a potent inhibitor of tau protein aggregation in Alzheimer’s disease.

### Resveratrol

Resveratrol, a nonflavonoid polyphenol phytoalexin, is broadly presented in grapes, giant knotweed, peanuts, etc. (Tian and Liu, 2020). Resveratrol is also enriched in the root of *Polygonum cuspidatum*, a well-known traditional Chinese medicine (Hu and Li, 2019). It is quite famous for various biological effects, including antioxidative, anti-tumor, anti-inflammation, anti-fibrosis, and neuroprotective activities (Parsamanesh et al., 2021; Uddin et al., 2021). Despite its potential for these effects on multiple diseases, the clinical usage of resveratrol is limited due to its pharmaceutical limitations, such as low bioavailability and poor bioavailability (Robertson et al., 2022).

Primary microglia-derived exosomes with encapsulated resveratrol (Exo-Res) have been shown to enhance the solubility and stability of resveratrol both *in vivo and in vitro* (Fan et al., 2020). A recent study supported that Exo-Res could increase muscle tension in hind limbs and improve foot functional movements in spinal cord injury rats. Furthermore, Exo-Res significantly inhibited apoptosis-related proteins caspase-3 and TUNEL expressions, accompanied by increasing autophagy-related proteins LC3B and Beclin-1 levels and p-PI3K expression in spinal cord injury rats (Fan et al., 2020). These findings suggested that Exo-Res has the potential for promoting motor functional recovery in spinal cord injury by activating autophagy and inhibiting apoptosis via the PI3K pathway. Nevertheless, the mechanism of autophagy that inhibits apoptosis has not been fully clarified. The release and targeting of resveratrol loaded in exosomes both *in vivo and in vitro* needs to be further studied.

### Norcantharidin

Norcantharidin (NCTD), a derivative of Cantharidin isolated from the dried body of *Mylabris phalerata Pallas*, has various pharmacological activities, including anti-tumor, anti-inflammatory, and anti-fibrosis properties (Zhou et al., 2020). Norcantharidin has been used to treat lung, breast, bladder, hepatic carcinoma, and prostate cancers (Pan et al., 2020). However, the clinical usage of Norcantharidin is restricted due to its poor water solubility, low tumor-targeting efficiency, and short half-life (Chi et al., 2019).

Bone mesenchymal stem cell-derived exosomes (BMSC-Exos) were used as drug carriers to encase NCTD (BMSC-Exos-NCTD), and its potential therapeutic effects against hepatocellular carcinoma (HCC) were explored (Liang Y. et al., 2021). The drug release study showed the release of NCTD was continuous and slow *in vitro* after it was packaged into BMSC-Exos. Compared with free NCTD, BMSC-Exos-NCTD significantly facilitated cellular uptake, induced cell cycle arrest in the G2 phase, inhibited tumor cell proliferation, decreased cell migration and invasion, and induced apoptosis in HepG2 cells. Interestingly, BMSC-Exos also inhibited HepG2 cell proliferation but was weaker than that of BMSC-Exos-NCTD treatment. Besides, BMSC-Exos-NCTD showed more obvious tumor inhibition effects *in vivo* than NCTD treatment alone, with no obvious tissue damage in the liver and kidney. The fluorescence intensity of ROS in the NCTD group was enhanced but weakened in the BMSC-Exos-NCTD group in the normal liver cell line L02. Importantly, Cy5.5, a fluorescent probe used to label BMS-Exos, was only concentrated in the liver tissues, while BMS-Exos-Cy5.5 enriched in liver tissues and tumor areas, especially in tumor areas, indicating that BMSC-Exos exhibit a homing effect on the tumor sites of HCC mice(Liang L. et al., 2021). Collectively, BMSC-Exos can be used as safe and effective drug-delivery carriers for HCC therapy. BMSC-Exos-NCTD has beneficial effects on anti-tumor and repairing liver cells in HCC without obvious toxicity. The mechanism of BMSC-Exos-NCTD on hepatocyte repairing may be related to increase cellular proliferation and inhibit the oxidation of liver cells. However, the underlying mechanisms of BMSC-Exos and BMSC-Exos-NCTD on HCC therapy have not been fully explained, hoping to be explored in future studies.

## Interplay between exosomes and TCM syndromes

Studies associated with TCM syndromes and exosomes have attracted more and more researchers’ attention. TCM syndrome, also known as “zheng” in Chinese, is a vital part of TCM theory and an abstract generalization of the pathological changes of a disease at a certain phase, which reveals inherent pathological variations of signs and symptoms (Su et al., 2014). In the clinical practice of TCM, TCM syndrome is of great significance for identifying human body patterns and guiding TCM clinicians to conduct an individual diagnosis and treatment with TCM herbs. Although there are few studies on TCM syndromes and exosomes, exosomes may provide a novel insight into TCM syndromes. Studies associated with TCM syndromes and exosomes are presented in Table 4.

**TABLE 4:** The interplay between Exosomes and traditional Chinese medicine syndrome.

Study ID	Aims of study	N	Subjects	TCM syndromes	Characteristics of exosomes					Results	Potential mechanisms
					Source	Isolation procedure	Morphology	Size	Membrane surface markers		
He et al. (2021)	To explore the pathogenesis of T2DM intestinal damp-heat syndrome from the perspective of exosomal miRNA	24	T2DM patients	Intestinal damp-heat syndrome	Saliva	ExoRNeasy Serum/Plasma Maxi Kit	Not reported	Not reported	Not reported	The levels of exosomal hsa-miR-9-5p, hsa-miR-150-5p, and hsa-miR-216b-5p were increased	These exosomes are primarily involved in cell development, body metabolism, TGF-β, and ErbB signaling pathways
You et al. (2021)	To evaluate the possibility of the location of a miRNA in plasma exosomes in patients with CAG of Pi-qi deficiency syndrome	5	Patients with CAG	Pi-qi-deficiency syndrome	Plasma	Using the ExoQuick exosome precipitation solution	Not reported	Not reported	Not reported	The levels of exosomal miRNA-122-5p kept higher expression both in leukocytes and serums	Exosomal miRNA-122-5p regulated leukocyte proliferation by regulating the TGF-β, signaling and chronic myeloid leukemia
Wang Y. et al. (2021)	To explore the role and mechanism of exosomes in spleen deficiency syndrome internal environment on HCC mice progression	40	HCC mice	Spleen deficiency syndrome	Serum	Ultracentrifugation	Spherical, membrane-bound vesicles	43–68 nm	Not reported	The level of serum exosomal CTLA-4 was increased	Exosomal CTLA-4 promotes the proliferation, self-renewal, and metastasis of hepatocellular carcinoma by regulating the PTEN/CD44 pathway

CAG, chronic atrophic gastritis; CTLA-4, cytotoxic T lymphocyte antigen 4; HCC, hepatocellular carcinoma; PTEN, phosphatase and tensin homolog; T2DM, type 2 diabetes mellitus.TGF-β, transforming growth factor-beta.

### Intestinal damp-heat syndrome

He et al. initiated a controlled study to explore the pathogenesis of type 2 diabetes mellitus (T2DM) intestinal damp-heat syndrome (IDHS) from the perspective of exosomal miRNA, which enrolled 24 patients with IDHS of T2DM and 24 healthy people (He et al., 2021). According to the results of chIp-sequencing and qPCR verification, exosomal hsa-miR-9-5p, hsa-miR-150-5p, and hsa-miR-216b-5p were significantly increased in the IDHS patients compared with the healthy group. These exosomal miRNAs are mainly enriched in cell development, body metabolism, and TGF-β signaling pathways. miR-9-5p exhibits effects on angiogenesis, anti-inflammation, and antioxidation. miR-9-5p inhibitor inhibited insulin release and triggered oxidative stress in streptozocin-induced INS-l cells (Ma et al., 2021). Exosomal miR-150-5p secreted by renal tubular epithelial cells activated fibroblasts and aggravated renal fibrosis (Zhou X. et al., 2021). Silencing of miR-150-5p played a renoprotective role in diabetic kidney disease (Dong W. et al., 2021b). The activation of the TGF-β signaling pathway contributes to metabolic disorders and fibrosis (Hou et al., 2018; Ji et al., 2022). Collectively, we infer that these exosomes may involve in oxidative stress, metabolism, and fibrosis via these signaling pathways in the T2DM patients with IDHS. However, the study’s findings should be interpreted cautiously because of a limited number of patients, necessitating more large-scale, multi-center, and high-quality clinical investigations to verify. Besides, the effects and potential mechanisms of these exosomal miRNA on T2DM by regulating the pathways mentioned above in the internal environment of intestinal damp-heat remain unknown, needing further investigation *in vivo*.

### Pi Qi deficiency syndrome

You et al. reported a study to investigate the miRNA-gene interactions underlying leukocyte functions and the potential serum biomarkers in patients with chronic atrophic gastritis (CAG) of Pi-qi deficiency syndrome (PQDS) (You et al., 2021). Ninety-nine differential miRNAs were found in the serums between the CAG patients with PQDS and healthy individuals. Among them, the hsa-miR-122-5p was the common differential miRNA in the leukocytes and serums of the CAG patients with PQDS. The hsa-miRNA-122-5p was loaded in human plasma exosomes and delivered to recipient cells throughout the body. Interestingly, exosomal miRNA-122-5p kept higher expression both in leukocytes and serums, indicating that the hsa-miR-122-5p may be a potential biomarker for CAG patients with PQDS. Furthermore, the target genes of the exosomal hsa-miR-122-5p were enrichment in the transforming growth factor-beta signaling and chronic myeloid leukemia, hinting it might have potential roles in the regulation of leukocyte proliferation in CAG patients with PQDS. However, the conclusions should be interpreted cautiously because only five patients were enrolled, wanting further studies with large-scale, multi-center, and high-quality to strengthen the findings. The roles and underlying mechanisms of exosomal miR-122-5p in CAG with PQDS also require exploration in subsequent studies.

### Spleen deficiency syndrome

You et al. (Wang Y. et al., 2021) conducted an experimental study to explore the role and mechanism of exosomes in spleen deficiency syndrome (SDS) internal environment on hepatocellular carcinoma (HCC) mice progression. It was found that the degree of malignancy of tumor tissue was higher in the HCC-SDS group than in the HCC group. Compared with HCC mice, the serum exosomal protein expressions of cytotoxic T lymphocyte antigen 4 (CTLA-4), programmed cell death protein 1 (PD-1), phosphatase and tensin homolog (PTEN), and AKT were higher in HCC-SDS mice. HepG2 cells treated with HCC-SDS mice serum had a stronger cell proliferation, and migration and invasion ability, while the CTLA-4 inhibitors could reverse these changes, indicating spleen deficiency may boost the occurrence and development of HCC via exosomal CTLA-4. It has been reported that the PTEN/CD44 involved tumor initiation, invasion, and metastasis (Luongo et al., 2019). The HCC-SDS group had significantly higher levels of CTLA-4 and CD44, and PTEN in the liver tissues compared with the HCC group. These results suggest that in the internal environment of spleen deficiency, exosomal CTLA-4 promotes the proliferation, self-renewal, and metastasis of HCC, likely associated with regulating the PTEN/CD44 pathway. However, the molecular mechanism of how CTLA-4 regulates the PTEN/CD44 pathway remains unclear. Although these findings may provide a new therapeutic target for patients with liver cancer, the conversion between animal experiments and clinical trials needs further exploration.

### Challenges and future prospects

**FIGURE 2:**
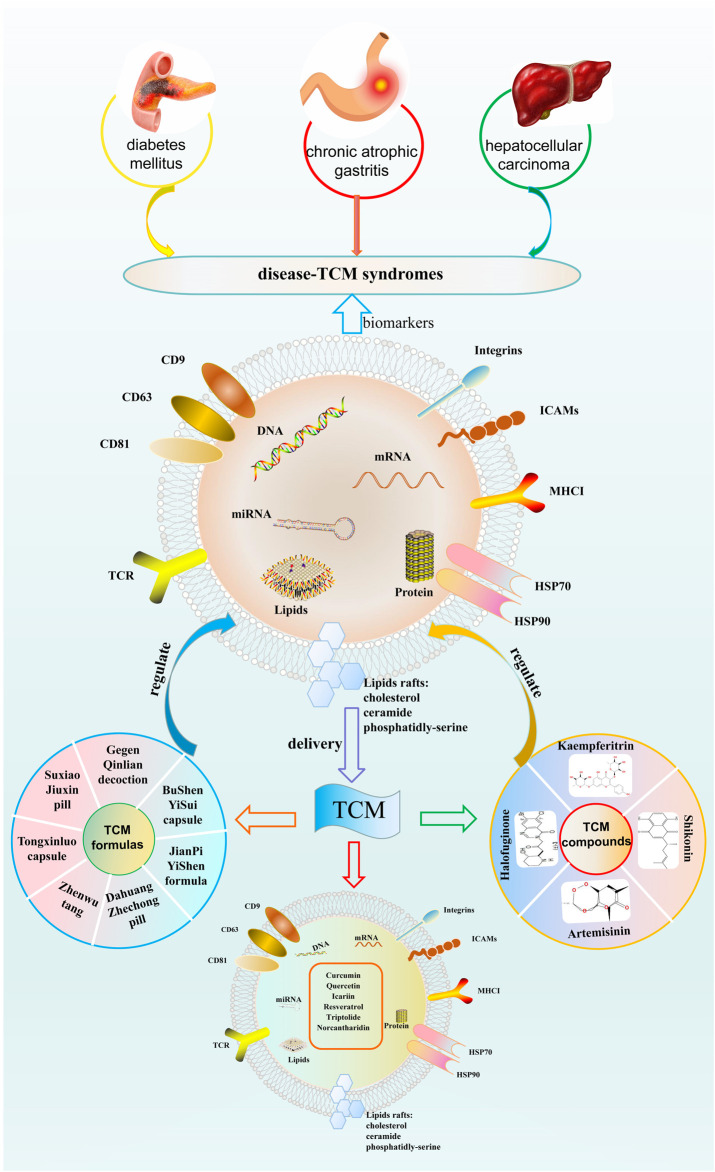
The interaction between exosomes and traditional Chinese medicine. TCM, traditional Chinese medicine.

TCM is a treasure of Chinese traditional culture, with thousands of years in clinical practice and development, which has accumulated abundant clinical experience and systematic theories. Nowadays, the modernization of TCM and the rapid iteration of systems biology provide an unprecedented opportunity to understand the science of TCM better. To our knowledge, this is the first review that presents a novel insight into TCM from the perspective of exosomes. Exosomes are important in delivering compounds isolated from TCM to target cells or tissues. TCM formulas and compounds isolated from TCM have beneficial effects on multiple disorders by regulating exosomes. Moreover, exosomes may be the potential biomarkers for TCM syndromes, providing strategies for TCM treatment. The interaction between exosomes and TCM is shown in Figure 2.

Recently, the studies of the interplay between exosomes and TCM have increased gradually, indicating this research field is getting more and more attention. However, there are still several obstacles to studying exosomes and TCM.

Firstly, studies of the interplay between exosomes and TCM are preliminary. Most studies have not elucidated the underlying mechanisms of TCM formulas and compounds isolated from TCM against diseases via regulating exosomes. More thorough understanding and research are still required both *in vivo and in vitro*. Moreover, the findings of clinical studies should be interpreted cautiously due to a limited number of subjects and poor study design. Large-sample, multicentre, high-quality, and well-designed clinical studies should be registered to achieve convincing results.

Secondly, the compositions of TCM formulas or compounds isolated from TCM are quite complex, with hundreds if not thousands of constituents. The various components of TCM can affect organisms via many biological reactions; however, this diversity may contribute to different active ingredients in TCM being synergistic, enhancing, and antagonistic (Wang A. et al., 2022). It is challenging to identify the effects and mechanisms of TCMs on modulating exosomes, highlighting that exploring the pathways and targets of each component alone and in different combinations is necessary.

Thirdly, there are still challenges with exosomes in studying TCM. Exosomes, as delivery vehicles, carry multiple intracellular signals, such as nucleic acids and proteins, which may play roles in the pathogenesis and progression of diseases. For example, BMSC-Exos could inhibit HepG2 cell proliferation (Liang L. et al., 2021). But the functions and mechanisms of exosomes on illness remain unclear in many studies that evaluated the effects of compounds-containing exosomes against disease; hoping the exosomes group should be considered in further studies both *in vivo and in vitro*. The purification and isolation techniques of exosomes are focused on the Manufacturer’s kit and ultracentrifugation (Tables 1–4). However, the results of the Manufacturer’s kit are easily affected by the laboratory environment and extractive technique. Ultracentrifugation has low yields, which can even damage exosomes due to the centrifugal forces applied to the vesicles (Massey et al., 2021). Other isolation methods, such as ultrafiltration, microfluidics-based isolation, and immunoaffinity capture, may obtain higher quality results. Besides, co-incubation was the most common strategy for cargos incorporated into exosomes and cargos-containing exosomes’ cellular uptake by recipient cells (Table 3). Although co-incubation is perhaps the simplest method to incorporate therapeutic agents into exosomes or target recipient cells, it is affected by multiple factors, such as temperature and humidity (Massey et al., 2021). Electroporation, sonication, freeze-thaw cycling, and chemical transfection are also methods for therapeutic loading in exosomes. However, the advantages and disadvantages of these methods have not been fully clarified, which may be a direction worthy of exploring.

Moreover, TCM syndrome contains a group of clinical symptoms with complicated pathological mechanisms. However, the potential mechanisms of exosomes on diseases in the internal environment of TCM syndrome are not well understood. Besides, the studies that investigated exosomes as biomarkers for TCM syndromes only focused on the expression of exosomes, which barely further validated their sensitivity and specificity. It is necessary to find an appropriate breakthrough point on the potential mechanisms and establish reasonable diagnostic exosomal models of TCM syndromes based on multi-omics technologies, bioinformatics analysis, and artificial intelligence. Importantly, further validation in clinical trials with large-sample and multicentre is indispensable.

## Conclusion

The review provides novel perspectives on the interplay between exosomes and TCM. TCM formulas and compounds isolated from TCM as exosome modulators have beneficial effects on multiple disorders, such as tumors, kidney diseases, and hepatic disease, which may involve cells proliferation inhibition, anti-inflammation, anti-oxidation, and attenuating fibrosis. Exosomes, a natural delivery system, play important roles in delivering compounds isolated from TCM to target cells or tissues. Moreover, exosomes may be the potential biomarkers for TCM syndromes, providing strategies for TCM treatment.
