# Supplementary material for: Exosomes: A novel insight into traditional Chinese medicine
Source: Front Pharmacol. 2022 Aug 29;13:844782. doi: 10.3389/fphar.2022.844782 (PMC9465299; doi:10.3389/fphar.2022.844782)
Supplement: Supplementary file 1 [file DataSheet1.DOCX]

**Exosomes:** **A** **Novel Insight into Traditional Chinese Medicine**

Chao Mo ^#^, Jie Zhao ^#^, Jingyan Liang , Huiling Wang , Yu Chen , Guodong Huang *

Supplementary appendix to the manuscript

Supplementary File 1

## Search strategy

| **1. Pubmed** |
| --- |
| #1 "Exosomes"[Mesh Terms]  #2 Exosome[Title/Abstract]  #3 Endosomes[Text Word]  #4 Exosome Multienzyme Ribonuclease Complex[Text Word]  #5 Cell-Derived Microparticles[Text Word]  #6 Secretory Vesicles[Text Word]  #7 "Extracellular Vesicles"[Mesh]  #8 Extracellular Vesicle[Title/Abstract]  #9 Vesicle, Extracellular[Text Word]  #10 Exovesicle[Text Word]  #11 #1 OR #2 OR #3 OR #4 OR #5 OR #6 OR #7 OR #8 OR #9 OR #10  #12 "Medicine, Chinese Traditional"[MeSH Terms]  #13 Traditional Chinese Medicine[Title/Abstract]  #14 Chinese Medicine, Traditional[Text Word]  #15 Chinese Traditional Medicine[Text Word]  #16 Traditional Medicine, Chinese[Text Word]  #17 Chinese herbal medicine [Text Word]  #18 "Drugs, Chinese Herbal"[MeSH Terms]  #19 Drugs, Chinese Herbal[Title/Abstract]  #20 Chinese Drugs, Plant[Text Word]  #21 Chinese Herbal Drugs[Text Word]  #22 Herbal Drugs, Chinese[Text Word]  #23 Plant Extracts, Chinese[Text Word]  #24 Chinese Plant Extracts[Text Word]  #25 Extracts, Chinese Plant[Text Word]  #26 "Natural product"[MeSH Terms]  #27 Natural product[Title/Abstract]  #28 Traditional Chiese medicine syndrome[Text Word]  #29 TCM syndrome[Text Word]  #30 #12 OR #13 OR #14 OR #15 OR #16 OR #17 OR #18 OR #19 OR #20 OR #21 OR #22 OR #23 OR #24 OR #25 OR #26 OR #27 OR #28 OR #29  #31 #11 AND #30 |
| **2.** **Embase** |
| #1 'Exosomes'/exp  #2 'Exosome':ab,ti  #3 'Endosomes':ab,ti  #4 'Exosome Multienzyme Ribonuclease Complex':ab,ti  #5 'Cell-Derived Microparticles':ab,ti  #6 'Secretory Vesicles':ab,ti  #7 'Extracellular Vesicles'/exp  #8 'Extracellular Vesicle':ab,ti  #9 'Vesicle, Extracellular':ab,ti  #10 'Exovesicle':ab,ti  #11 #1 OR #2 OR #3 OR #4 OR #5 OR #6 OR #7 OR #8 OR #9 OR #10  #12 'Medicine, chinese traditional'/exp  #13 'Traditional chinese medicine':ab,ti  #14 'Chinese Medicine, Traditional':ab,ti  #15 'Chinese Traditional Medicine':ab,ti  #16 'Traditional Medicine, Chinese':ab,ti  #17 'Chinese herbal medicine':ab,ti  #18 'Drugs, Chinese Herbal'/exp  #19 'Drugs, Chinese Herbal':ab,ti  #20 'Chinese Drugs, Plant':ab,ti  #21 'Chinese Herbal Drugs':ab,ti  #22 'Herbal Drugs, Chinese':ab,ti  #23 'Plant Extracts, Chinese':ab,ti  #24 'Chinese Plant Extracts':ab,ti  #25 'Extracts, Chinese Plant':ab,ti  #26 'Natural product'/exp  #27 'Natural product':ab,ti  #28 'Traditional Chiese medicine syndrome':ab,ti  #29 'TCM syndrome':ab,ti  #30 #12 OR #13 OR #14 OR #15 OR #16 OR #17 OR #18 OR #19 OR #20 OR #21 OR #22 OR #23 OR #24 OR #25 OR #26 OR #27 OR #28 OR #29  #31 #11 AND #30 |
| **3.** **Cochrane Library** |
| #1 MeSH descriptor: [Exosomes] explode all trees  #2 Exosome: ti,ab,kw  #3 Endosomes: ti,ab,kw  #4 Exosome Multienzyme Ribonuclease Complex: ti,ab,kw  #5 Cell-Derived Microparticles: ti,ab,kw  #6 Secretory Vesicles: ti,ab,kw  #7 MeSH descriptor: [Extracellular Vesicles] explode all trees  #8 Extracellular Vesicle: ti,ab,kw  #9 Vesicle, Extracellular: ti,ab,kw  #10 Exovesicle: ti,ab,kw  #11 #1 OR #2 OR #3 OR #4 OR #5 OR #6 OR #7 OR #8 OR #9 OR #10  #12 MeSH descriptor: [medicine, chinese traditional] explode all trees  #13 Traditional chinese medicine: ti,ab,kw  #14 Chinese Medicine, Traditional: ti,ab,kw  #15 Chinese Traditional Medicine: ti,ab,kw  #16 Traditional Medicine, Chinese: ti,ab,kw  #17 Chinese herbal medicine: ti,ab,kw  #18 MeSH descriptor: [Drugs, Chinese Herbal] explode all trees  #19 Drugs, Chinese Herbal: ti,ab,kw  #20 Chinese Drugs, Plant: ti,ab,kw  #21 Chinese Herbal Drugs: ti,ab,kw  #22 Herbal Drugs, Chinese: ti,ab,kw  #23 Plant Extracts, Chinese: ti,ab,kw  #24 Chinese Plant Extracts: ti,ab,kw  #25 Extracts, Chinese Plant: ti,ab,kw  #26 MeSH descriptor: [natural product] explode all trees  #27 Natural product: ti,ab,kw  #28 Traditional Chiese medicine syndrome : ti,ab,kw  #29 TCM syndrom: ti,ab,kw  #30 #12 OR #13 OR #14 OR #15 OR #16 OR #17 OR #18 OR #19 OR #20 OR #21 OR #22 OR #23 OR #24 OR #25 OR #26 OR #27 OR #28 OR #29  #31 #11 AND #30 |
| **4. Web of Science** |
| #1 TS= (Exosomes OR Secretory Vesicle OR Cell-Derived Microparticles OR Exosome Multienzyme Ribonuclease Complex OR Endosomes OR Extracellular Vesicles OR Vesicle, Extracellular OR Exovesicle )  #2 TS= ('Medicine, chinese traditional' OR 'Traditional Medicine, Chinese' OR Chinese Traditional Medicine OR 'Chinese Medicine, Traditional' OR Chinese herbal medicine OR 'Drugs, Chinese Herbal' OR 'Chinese Drugs, Plant' OR 'Chinese Herbal Drugs' OR 'Herbal Drugs, Chinese' OR 'Plant Extracts, Chinese' OR 'Chinese Plant Extracts' OR 'Extracts, Chinese Plant' OR natural product OR traditional Chiese medicine syndrome OR TCM syndrome)"  #3 #1 AND #2 |
| **5. Clinical Trials.gov** |
| Advanced search:  1. Other terms= ("Exosomes" OR "Secretory Vesicle" OR "Cell-Derived Microparticles" OR "Exosome Multienzyme Ribonuclease Complex" OR "Endosomes OR Extracellular Vesicles" OR "Vesicle, Extracellular" OR "Exovesicle") AND ("Medicine, chinese traditional" OR "Traditional Medicine, Chinese" OR "Chinese Traditional Medicine" OR "Chinese Medicine, Traditional" OR "Chinese herbal medicine" OR "Drugs, Chinese Herbal" OR "Chinese Drugs, Plant" OR "Chinese Herbal Drugs" OR "Herbal Drugs, Chinese"OR "Plant Extracts, Chinese" OR "Chinese Plant Extracts" OR "Extracts, Chinese Plant"OR "natural product" OR "traditional Chiese medicine syndrome" OR "TCM syndrome")  2. Study type= All studies  3. Study Results=All studies |
